# Kurarinone ameliorates intestinal mucosal inflammation via regulating T cell immunity

**DOI:** 10.3389/fimmu.2025.1587479

**Published:** 2025-05-01

**Authors:** Yan Pan, Bolin Deng, Tingting Wang, Zhou Zhou, Jinxia Wang, Caiping Gao, Chong He

**Affiliations:** ^1^ Department of Gastroenterology, Sichuan Provincial People’s Hospital, University of Electronic Science and Technology of China, Chengdu, China; ^2^ Translational Clinical Immunology Key Laboratory of Sichuan Province, Sichuan Provincial People’s Hospital, University of Electronic Science and Technology of China, Chengdu, China

**Keywords:** kurarinone, experimental colitis, inflammatory bowel disease, intestinal epithelial barrier, Th17 cells

## Abstract

**Background:**

Inflammatory bowel disease (IBD) has become an increasingly significant global health concern, imposing substantial economic and psychological burdens on society and public health systems. Herbal medicines, which have shown promise in alleviating IBD symptoms and promoting remission through mechanisms such as immune regulation and anti-inflammatory effects, are gaining increasing attention. Kurarinone (KAR) is a major component of the dried roots of *Sophora flavescens*, which exhibits a range of pharmacological activities, including antioxidant and anti-inflammatory effects. However, research on the therapeutic potential of KAR in IBD, particularly its effect on intestinal mucosal inflammation, remains limited.

**Methods:**

Colitis was induced by trinitrobenzene sulfonic acid (TNBS) in mice and KAR was intraperitoneally given. Hematoxylin and eosin staining, flow cytometry, and immunofluorescence were used for mucosal inflammation evaluation. Changes in gut microbiota were assessed using 16S rRNA sequencing. RNA sequencing was performed to screen for KAR’s therapeutic targets, which was verified by *in vitro* T cell culture.

**Results:**

We demonstrated that administration of KAR resulted in a mitigated colonic tissue damage in mice with TNBS-induced colitis and decreased the infiltration of inflammatory cells, including monocytes/macrophages, neutrophils, and T lymphocytes. Moreover, KAR protected TNBS-insulted mice from colonic goblet cell loss and tight junction destruction. Furthermore, KAR treatment led to the restoration of the gut microbiota to a more normal composition. Mechanistically, KAR suppressed T helper (Th) 17 cell response but facilitated interleukin (IL)-10 production via Blimp-1.

**Conclusion:**

Our study investigated the impact of KAR on mice with TNBS-induced colitis and elucidated its underlying mechanisms, thereby uncovering novel possibilities for clinical interventions in IBD.

## Introduction

Inflammatory bowel disease (IBD) is a chronic and relapsing non-specific inflammatory disorder of the gastrointestinal tract, encompassing two main types: ulcerative colitis (UC) and Crohn’s disease (CD). IBD has become an increasingly significant global health concern, with a rising prevalence observed in both developed and developing countries (1, 2). The exact cause of IBD remains unknown, but it is believed to result from a complex interplay of genetic, environmental, and immunological factors (3). The disease significantly impacts the quality of life of affected individuals, imposing substantial economic and psychological burdens on society and public health systems (4).

Traditional therapies for IBD primarily include sulfasalazine, 5-aminosalicylic acid (5-ASA) agents, corticosteroids, and immunosuppressive agents, which mainly target intestinal inflammation and immune responses (5). However, these conventional treatments often fall short in achieving satisfactory therapeutic outcomes, with many patients experiencing incomplete remission or relapse. Given these limitations, the quest for more effective therapeutic agents continues to be a paramount goal for researchers. Modern pharmacological research has confirmed that traditional Chinese medicine (TCM) is a vast treasure trove of medical knowledge. Numerous effective components have been extracted from Chinese herbal medicine and widely used in clinical treatments (6). The use of herbal medicines for the treatment of IBD has gained increasing attention in recent years (7). Traditional herbal remedies have been used for centuries in various cultures and have shown promising effects in alleviating IBD symptoms and promoting remission (6).

*Sophora flavescens Aiton*, commonly known as “Kushen” in traditional Chinese medicine, has a history of several thousand years in treating intestinal symptoms such as abdominal pain and diarrhea (8). Kurarinone (KAR) is a major component of the dried roots of *Sophora flavescens* and is found in various other plants. It exhibits a range of pharmacological activities, including anticancer, antifungal, antibacterial, antiviral, neuroprotective, antioxidant, and anti-inflammatory effects (9–14). Previous studies have shown that KAR can activate the KEAP1/Nrf2 pathway to induce heme oxygenase-1 (HO-1) expression, inhibit interleukin (IL)-1β inflammation in RAW264.7 macrophages stimulated by lipopolysaccharides, and induce the expression of inducible nitric oxide synthase (iNOS), thereby exerting immunosuppressive effects (15). Jia et al. discovered that KAR upregulates the expression of insulin-like growth factor 1 (IGF1) in heme-treated HMC3 cells and activates the PI3K/Akt signaling pathway, promoting M2 polarization of microglia and inhibiting heme-induced neuroinflammation and neurotoxicity mediated by M1 polarization of microglia (10). These findings demonstrate the potent anti-inflammatory and immunomodulatory effects of KAR. Furthermore, KAR has been shown to mitigate oxidative stress and apoptosis in human ovarian granulosa cells induced by H_2_O_2_ through upregulating the expression of IGF1 and activating the PI3K/Akt signaling pathway (11). These antioxidative and anti-apoptotic properties of KAR suggest its potential protective role in maintaining epithelial barrier function.

Despite the growing interest in the therapeutic potential of various compounds for IBD, research on the effects of KAR remains limited. This study aims to fill this gap by investigating the therapeutic potential of KAR in its overall efficacy in IBD and modulating intestinal mucosal inflammation, potentially offering new insights and treatment options for this challenging condition.

## Materials and methods

### Mice and trinitrobenzene sulfonic acid-induced colitis model

The Animal Care and Use Committee at Sichuan Provincial People’s Hospital approved all experimental procedures (No. 2020204), which adhered to the National Institutes of Health guidelines for animal care and use. Male C57BL/6 mice, aged 10–12 weeks, were obtained from Shanghai Model Organisms (Shanghai, China). Blimp-1 knockout (KO) mice were purchased from the Jackson Laboratory (Bar Harbor, Maine, USA). The mice were housed in a specific pathogen-free environment in our facility. Experimental colitis was induced as reported previously (16). Briefly, fasted mice received a rectal administration of TNBS dissolved in 50% ethanol, with a dosage of 2.0-2.5 mg. As described previously (17), the severity of colitis was assessed daily during the modeling period by monitoring weight and disease activity index (DAI) as follows: normal stools = 0, soft stools = 1, soft stools and slight bleeding = 2, loose stools and slight bleeding = 3, watery diarrhea or loose stools and gross bleeding = 4. Colonic pathological scoring was assessed as described previously (17). Briefly, a 0–4 grading scale was utilized, considering factors such as the percentage of colon affected by inflammation, the extent of crypt loss, the presence of lymphoid follicles, edema, erosions, and the density of inflammatory cell infiltration. The overall severity score was determined by summing the individual parameter scores. For the KAR+ TNBS group, TNBS-insulted mice received intraperitoneal administration of KAR (dissolved in dimethyl sulfoxide) from day 0 to day 7. The optimal dose of KAR (125 mg/kg/day) for the TNBS model has been established in our previous study (12). On day 7, all mice were euthanized by cervical dislocation, and colon samples were collected for further analysis. The control group consists of mice that only underwent the same procedural steps without the induction of colitis or administration of any therapeutic agents.

### Lamina propria leukocyte isolation

Lamina propria were obtained and LPL were purified as reported previously (16, 18, 19). Briefly, intestinal epithelial cells (IEC) were removed in ethylenediaminetetraacetic acid (EDTA) solution (5 mM EDTA, 10% fetal bovine serum (FBS) in Ca^2+^/Mg^2+^ free phosphate buffered saline (PBS)), and the residual tissues were subjected to enzymatic digestion (type IV collagenase, 1mg/ml, Sigma Aldrich, Burlington, MA, USA). After digestion, LPL in the supernatants were purified by gradient separation (40%/80% Percoll gradient).

### Intestinal permeability assay

To assess intestinal permeability, a previously described method was employed (17). Briefly, following a 6–8 hour period of food and water deprivation, mice were orally administered FD-4 (FITC-conjugated Dextran, FITC-Dextran, Sigma-Aldrich) at a dosage of 0.5 mg/kg body weight. After 4 hours, blood samples were obtained and the fluorescence intensity in the sera was measured.

### Flow cytometry

Flow cytometry analysis was conducted as reported previously (16–19). In brief, cells were stained for viability dye (LIVE/DEAD™ Fixable Near-IR Dead Cell Stain Kit, Invitrogen, Thermo Fisher Scientific, USA) and fluorochrome-conjugated antibodies (purchased from BioLegend) specific to the target markers for 30 minutes at 4 °C. Flow cytometric data were acquired using a BD FACSCanto II and analyzed with FlowJo version 10 for Windows (Tree Star, Ashland, OR, USA).

### Immunofluorescence

As reported previously (17, 19), 6 μm-thick frozen sections of colon tissue were prepared, which were then blocked with a solution containing 5% bovine serum albumin (BSA) and 0.5% Triton × 100 in PBS for 2 hours at room temperature. Next, the sections were incubated overnight at 4°C with the specified primary antibodies. Secondary antibodies were added and incubated with the colonic sections at room temperature for 2 hours. Finally, the slides were sealed with neutral resin and photographed for further analysis.

### Hematoxylin and eosin and periodic acid-Schiff staining

As reported previously (17), paraffin sections of the colon (4 μm) were stained following the instructions provided in the H&E staining kit (Servicebio, Wuhan, China) after dewaxing. To label the colonic goblet cells, PAS staining was performed on paraffin colon sections. After dewaxing and hydration, the sections were treated with periodic acid solution and then washed and incubated in Schiff’s reagent.

### 16s rRNA sequencing

DNA extraction from fecal samples involved thawing the samples at room temperature and collecting approximately 200 mg on a cotton swab, which was then placed in a centrifuge tube containing 1.2 ml of lysis buffer. After vortexing and centrifugation, the supernatants were transferred to a new tube and treated with proteinase K and AL buffer. Ethanol was added, and the mixture was loaded onto an adsorption column to collect microbial DNA. The DNA extraction quality was assessed using agarose gel electrophoresis. For polymerase chain reaction (PCR) amplification of the target fragment, primers specific to V3-V4 region were designed, along with sample-specific barcode sequences. This allowed for PCR amplification of the variable regions of the rRNA gene or specific gene fragments. Amplification products were purified and recovered using magnetic beads. After mixing the PCR products with the beads and subsequent adsorption, the supernatants were collected. Ethanol was added, and the supernatants were collected again. Finally, the PCR tubes were placed on a magnetic stand, and the supernatants were aspirated into a centrifuge tube. The amplification products were quantified using the Quant-iT PicoGreen dsDNA Assay Kit and a Microplate reader. Based on the fluorescence quantification results, the samples were mixed in the appropriate proportions for sequencing. Sequencing libraries were prepared using the Illumina TruSeq Nano DNA LT Library Prep Kit, and quality checks were performed using the Agilent Bioanalyzer. Once deemed satisfactory, the libraries were subjected to Illumina MiSeq sequencing.

### *In vitro* T cell differentiation and T cell suppression assay

As reported previously (20, 21), mouse naïve CD4^+^ T cells were isolated from the spleen and then were cultured with transforming growth factor (TGF)-β (10 ng/ml), IL-6 (30 ng/ml), anti-interferon (IFN)-γ (10 μg/ml), and anti-IL-4 (10 μg/ml) (referred to as Th17 polarizing condition) in the presence of plate-bound anti-CD3 plus anti-CD28. For T cell suppression assay, CD45.1 CD4^+^ T cells were labeled using a CellTrace Cell Proliferation Kit (Thermo Fisher Scientific, USA) according to the manufacturer’s instruction. Carboxyfluorescein succinimidyl ester (CFSE)-labeled CD45.1 CD4^+^ T cells were activated and cultured with the indicated CD45.2 Th17 cells for 5 days. After harvesting, the CFSE intensity was analyzed via flow cytometry by gating on CD45.1.

### Statistical analysis

Statistical analyses were performed with GraphPad Prism version 8.4 for Windows (GraphPad Software, San Diego, CA, USA). Unpaired Student’s t test (two-tailed) was applied for comparison between two groups were analyzed, and one-way analysis of variance (ANOVA) followed by Tukey’s multiple comparisons test was applied to analyze differences among three or more groups. Statistical significance was set at *p < 0.05.

## Results

### KAR ameliorates TNBS-induced colitis

**Figure 1:**
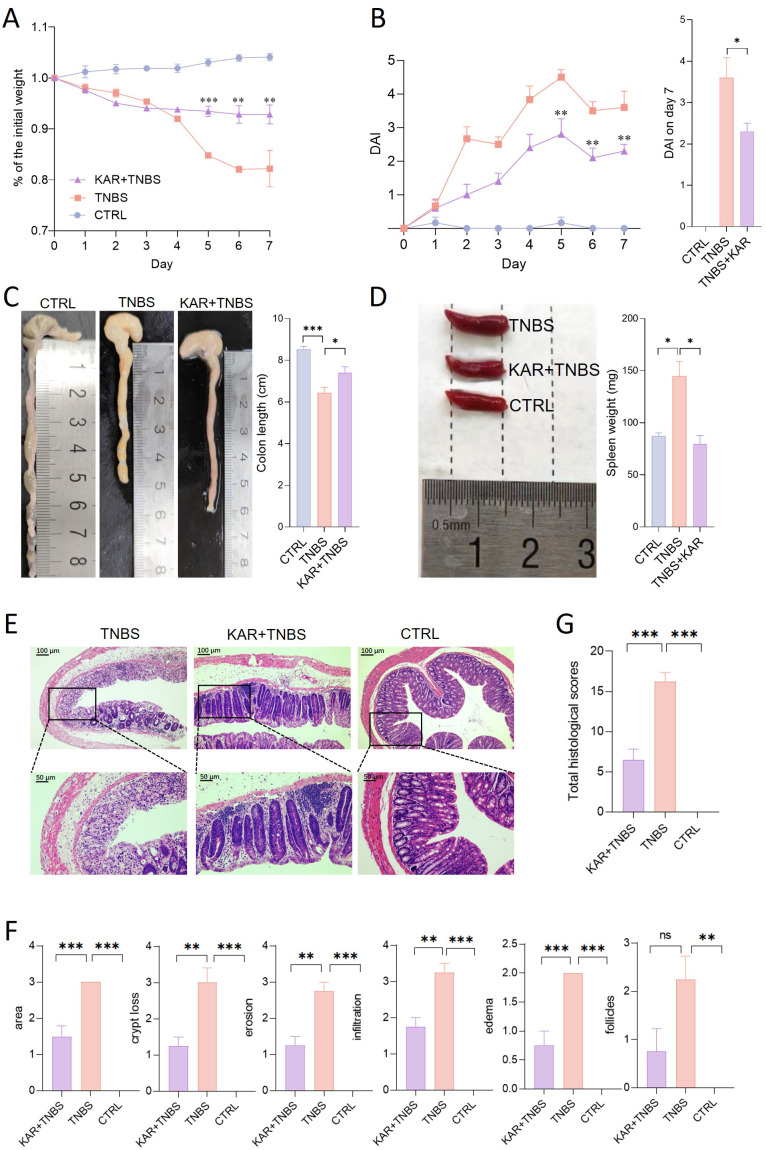
Kurarinone (KAR) ameliorates trinitrobenzene sulfonic acid (TNBS)-induced colitis. Colitis was induced by TNBS in mice. **(A)** Changes in body weight of TNBS-induced colitis mice. **(B)** Disease activity index (DAI) scores. **(A, B)** **p < 0.01; ***p < 0.001 versus the TNBS group. **(C)** Comparison of colon lengths and representative images. **(D)** Spleen weights and representative images. **(E)** Colon tissue histology stained with hematoxylin and eosin (H&E). **(F)** Colonic sections were scored on a scale of 0-4 based on the percentage of colon involvement by inflammation, percentage of crypt loss, presence of lymphoid follicles, edema, erosions, and density of infiltrating inflammatory cells. **(G)** Total histological scores were the sum of all sub-scores shown in **(F)**. Data are presented as mean ± SD. Representative data of three independent experiments are shown. N = 5 mice each group; *p < 0.05; **p < 0.01; ***p < 0.001; ns, no statistical difference. One-way analysis of variance (ANOVA) followed by Tukey's multiple comparisons test. TNBS+KAR group: TNBS-insulted mice treated with KAR; TNBS group: TNBS-insulted mice without treatment of KAR. CTRL: control mice without TNBS insults.

We employed a TNBS-induced colitis mouse model to investigate the therapeutic effects of KAR on colonic inflammation and potential mechanisms. The group treated with KAR (KAR+TNBS) exhibited significantly less weight loss compared to the non-treated TNBS group (TNBS group) (**Figure 1A**). DAI scores, used to assess the severity of colitis in mice, were noticeably lower in the KAR+TNBS group than in the TNBS group (**Figure 1B**). It was observed that the colons of the KAR+TNBS group were longer than those of the TNBS group (**Figure 1C**). The KAR+TNBS group also exhibited thinner colonic walls and less congestion, swelling, and necrosis compared to the TNBS group (**Figure 1C**). The KAR+TNBS group showed less pronounced splenomegaly than the TNBS group (**Figure 1D**). To further evaluate the severity of intestinal mucosal lesions, histological examination of colon sections stained with H&E revealed that the KAR+TNBS group exhibited better mucosal epithelial integrity, less reduction in goblet cell numbers, relatively preserved crypt structures, and only a minimal amount of lymphocyte infiltration compared to the TNBS group (**Figure 1E**). Pathological scoring of the inflammatory cell infiltration and tissue damage in the H&E -stained sections using a scoring scale showed significantly lower scores in the KAR+TNBS group than in the TNBS group (**Figures 1F, G**), indicating that colonic inflammation was milder in the KAR+TNBS group. These results demonstrate that KAR effectively alleviates TNBS-induced mucosal pathological damage in the colon of mice.

### KAR suppresses mucosal inflammatory cell infiltration in TNBS-induced colitis

**Figure 2:**
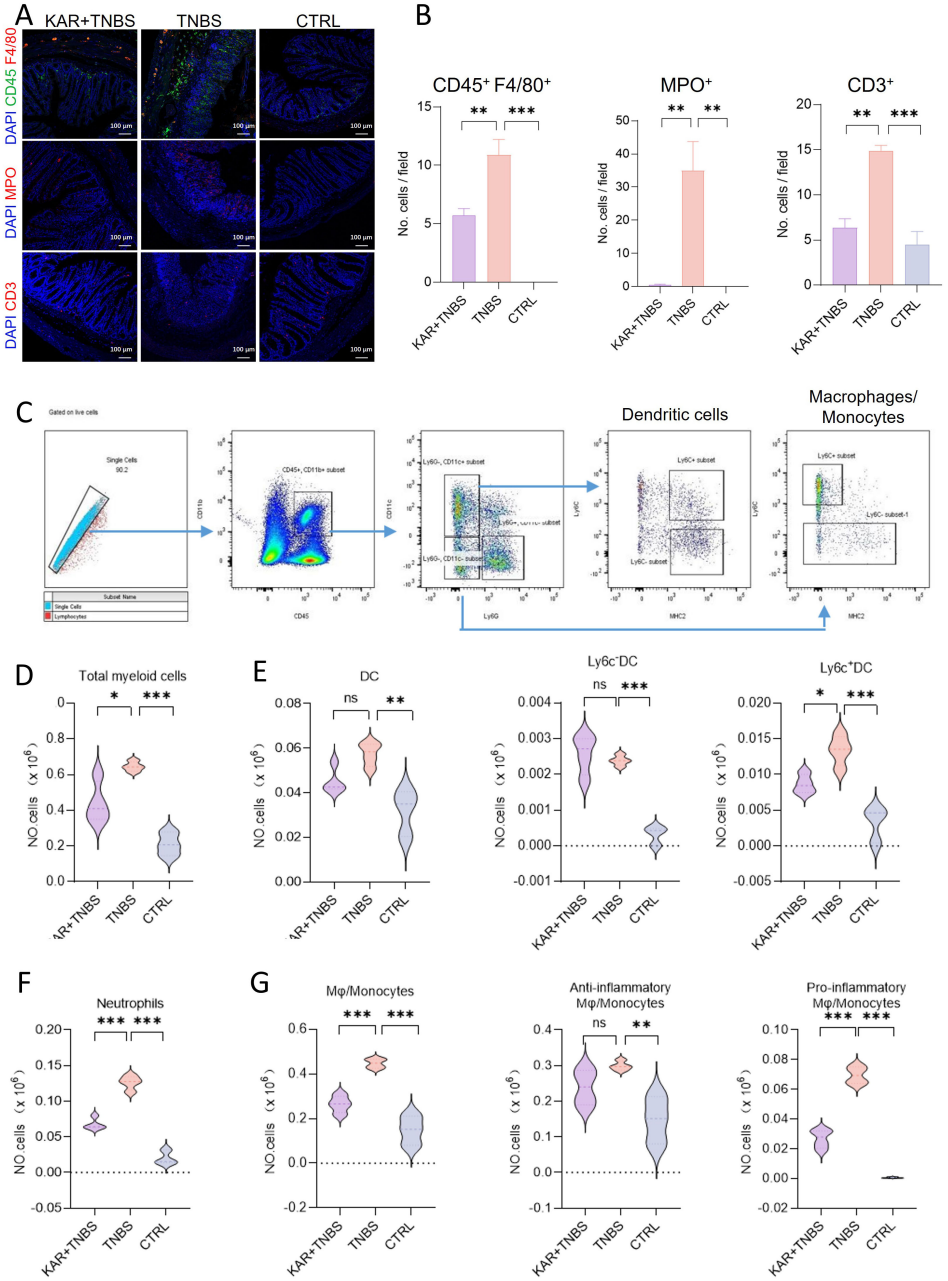
KAR suppresses inflammatory cell infiltration in the colon of mice with TNBS-induced colitis. Colon tissues were collected from indicated groups of mice (as described in **Figure 1**) and prepared for **(A, B)** immunofluorescence staining and **(C–G)** flow cytometry, respectively. **(A)** Immunofluorescence staining of monocytes/macrophages (CD45^+^ F4/80^+^, *upper*), neutrophils (MPO^+^, *middle*), and T cells (CD3^+^, *lower*). **(B)** Quantification of indicated cells. **(C)** Immune cell gating strategy in the colonic lamina propria. **(D–G)** Quantification of **(D)** total myeloid cells (CD45^+^ CD11b^+^), (**E**, *left*) total dendritic cells (DC, CD45^+^ CD11b^+^ Ly6G^-^ CD11c^+^), (**E**, *middle*) Ly6C^-^ DC (CD45^+^ CD11b^+^ Ly6G^-^ CD11c^+^ Ly6C^-^), (**E**, *right*) Ly6C^+^ DC (CD45^+^ CD11b^+^ Ly6G^-^ CD11c^+^ Ly6C^+^), **(F)** neutrophils (CD45^+^ CD11b^+^ Ly6G^+^), (**G**, *left*) total monocytes/macrophages (CD45^+^ CD11b^+^ CD11c^-^ Ly6G^-^), (**G**, *middle*) anti-inflammatory monocytes/macrophages (CD45^+^ CD11b^+^ CD11c^-^ Ly6G^-^ Ly6C^-^), and (**G**, *right*) pro-inflammatory monocytes/macrophages (CD45^+^ CD11b^+^ CD11c^-^ Ly6G^-^ Ly6C^+^ MHC II^-^). Data are presented as mean ± SD. Representative data of three independent experiments are shown. N = 5 mice each group; *p < 0.05; **p < 0.01; ***p < 0.001; ns, no statistical difference. One^-^way ANOVA followed by Tukey's multiple comparisons test.

There is a significant increase in various inflammatory cell infiltrates within the colonic lamina propria during colitis, which is a typical hallmark. TNBS-induced colitis is characterized by mucosal pro-inflammatory immune cell infiltration (16). To this end, we performed immunofluorescent staining using F4/80, myeloperoxidase (MPO), and CD3 markers to label macrophages, neutrophils, and T cells in the colon, respectively (**Figure 2A**). In the KAR+TNBS group, the infiltration of these three types of inflammatory cells in the colonic tissue was significantly lower than in the TNBS group (**Figure 2B**). Next, we further analyzed the immune cell profile in the lamina propria using flow cytometry (**Figure 2C**). The number of CD45^+^ CD11b^+^ cells (total myeloid cells) was significantly reduced after KAR treatment in colitis (**Figure 2D**). We further marked dendritic cells (Ly6G^−^ CD11c^+^), monocytes/macrophages (Ly6G^−^ CD11c^−^), and neutrophils (Ly6G^+^ CD11c^−^) from the total myeloid cells by labeling CD11c and Ly6G. The total number of dendritic cells also decreased after KAR treatment, but the difference was not statistically significant (**Figure 2E**), while the infiltration of neutrophils in TNBS mice was significantly reduced after KAR treatment (**Figure 2F**). Additionally, the infiltration of monocytes/macrophages (Ly6G^−^ CD11c^−^) in the lamina propria was significantly lower in the KAR+TNBS group compared to the TNBS group (**Figure 2G**). Dendritic cells and monocytes/macrophages can be further divided into pro-inflammatory and anti-inflammatory subtypes, which can be identified by specific surface markers (17, 22, 23). Compared to the TNBS group, the number of pro-inflammatory dendritic cell subsets (Ly6c^+^ MHCII^−^) in the KAR+TNBS group showed a significant decrease (**Figure 2E**). The anti-inflammatory subset (Ly6c^−^) displayed an upregulation trend, but without statistical significance (**Figure 2E**). The number of pro-inflammatory monocytes/macrophages (Ly6c^+^ MHCII^−^) in the KAR+TNBS group was significantly reduced compared to the TNBS group, while the anti-inflammatory subset (Ly6c^−^) also showed a downward trend in TNBS mice after KAR treatment, but without statistical significance (**Figure 2G**). These results confirm that KAR reduces mucosal inflammation in the colon of colitic mice.

### KAR maintains intestinal epithelial barrier function in TNBS-induced colitis

**Figure 3:**
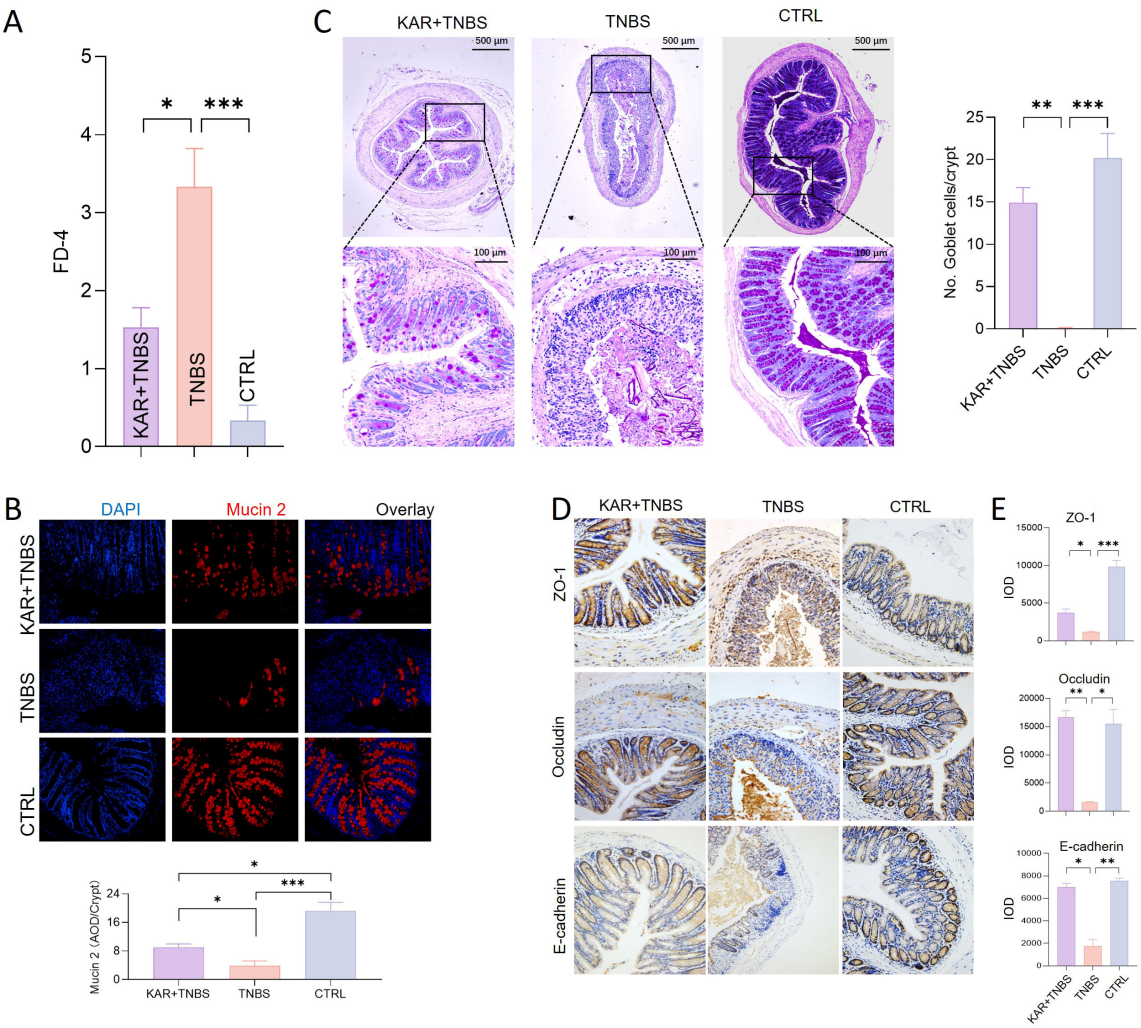
KAR maintains intestinal epithelial barrier function. Colitis was induced in mice by TNBS. **(A)** Intestinal permeability in all three groups was assessed on day 7. Fluorescence intensity of FD-4 in serum after 4 hours of FD-4 gavage. **(B)** Colonic sections were stained for Mucin 2 and DAPI. (**B**, *left*) Representative immunofluorescence images are shown. (**B**, *right*) Quantification of Mucin 2 expression was assessed by calculating the average optical density (AOD) of immunofluorescence signal. **(C)** Goblet cells were identified by periodic acid-schiff (PAS) staining. (**C**, *left*) Representative images are shown. (**C**, *right*) The number of goblet cells per crypt was assessed. **(D)** Immunohistochemical staining of tight junction proteins, including (*upper*) ZO-1, (*middle*) Occludin, and (*lower*) E-cadherin. Representative images are shown. **(E)** Quantitative analysis of colonic epithelial (*upper*) ZO-1, (*middle*) Occludin, and (*lower*) E-cadherin. IOD: integrated optical density, representing the cumulative light density. Data are presented as mean ± SD. Representative data of three independent experiments are shown. N = 5 mice each group; *p < 0.05; **p < 0.01; ***p < 0.001. One-way ANOVA followed by Tukey's multiple comparisons test.

The intestinal epithelial barrier is the first line of defense against harmful substances entering the internal environment. Impaired IEC barrier function in IBD is a hallmark of inflammation occurrence and development (18). Restoring the damaged intestinal epithelial barrier function can effectively alleviate intestinal inflammation (17). Therefore, we investigated whether KAR could improve the impaired IEC barrier function during colitis. Firstly, we started with the detection of IEC barrier permeability, showing that the serum FD-4 fluorescence intensity in the KAR+ TNBS group was significantly lower than that in the TNBS group (**Figure 3A**), which suggested that KAR could protect the integrity of the IEC barrier function. Secondly, the mucus barrier is an important component of the IEC barrier, and we examined the expression of Mucin 2 in each group of mice. As shown in **Figure 3B**, KAR significantly rescued the loss of Mucin 2 caused by TNBS modeling. Goblet cells, which secreted mucins, were also quantified used PAS staining and the number of goblet cells in the colonic mucosa of TNBS group mice was significantly lower than that in the control group, while it was significantly improved in the KAR+ TNBS group mice compared to the TNBS group mice (**Figure 3C**). These results suggest that KAR can protect goblet cells from damage, increase mucin secretion. The tight junctions adjacent to IEC constitute the physical barrier of the intestinal barrier. We found that the KAR treatment remarkably improved the damage of tight junctions ZO-1, Occludin, and E-cadherin in TNBS-induced colitis (**Figures 3D, E**). These data suggest that KAR protects the IEC barrier function during intestinal inflammation.

### The effect of KAR on gut microbiota during colitis

**Figure 4:**
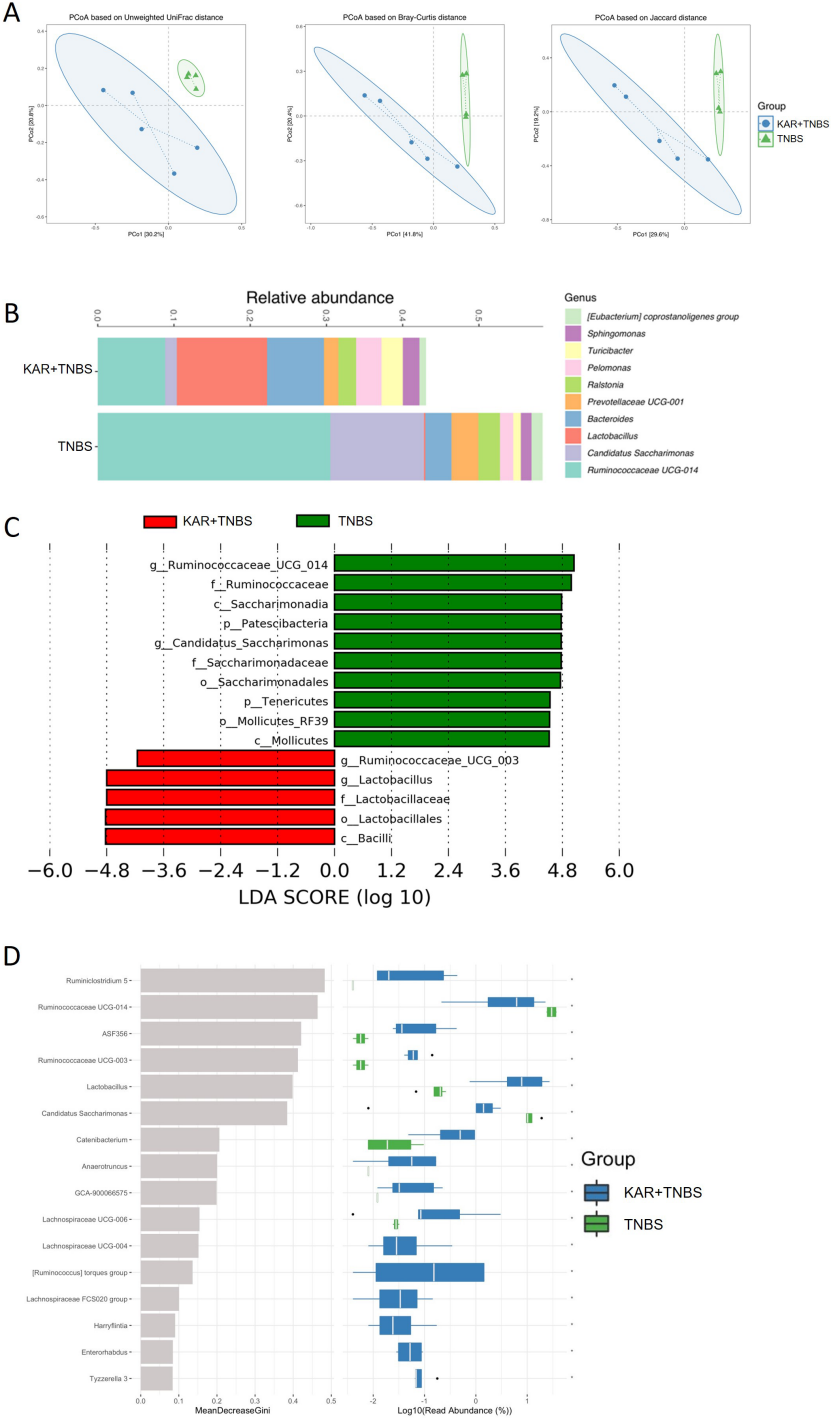
KAR regulates the imbalance of gut microbiota. Fecal samples were collected from KAR+TNBS and TNBS groups. Gut microbiota homeostasis was analyzed by 16S rRNA gene sequencing. **(A)** β diversity analysis. **(B)** The bar graph at the genus level of the intestinal microbiota based on 16S sequencing results. Different colors denote different taxa. **(C)** LefSe analysis. **(D)** Random forest analysis.

The occurrence of IBD often accompanies dysbiosis of the gut microbiota, and studies suggest that the gut microbiota may be an initiating factor in the development of IBD (24). As presented above, KAR could alleviate colitis, but its effects on the gut microbiota are still unknown. To this end, we employed 16S rRNA gene sequencing to investigate the changes in gut microbiota homeostasis in colitic mice treated with KAR. We analyzed the community structure of the gut microbiota. Using β diversity analysis (**Figure 4A**), we calculated the distances between samples in the TNBS and KAR+TNBS group using Unweighted Unifrac, Bray-Curtis, and Jaccard metrics. The samples within the KAR+TNBS group were closer to each other than to those within the TNBS group, and there were no overlapping samples between the two groups, indicating good repeatability within each group. The confidence ellipses of the KAR+TNBS and TNBS group showed clear deviation, suggesting differences in the gut microbiota community between the two groups. Subsequently, we performed permutational multivariate analysis of variance (PerMANOVA) to statistically analyze the distances between the KAR group and the TNBS group. The p-values based on Unweighted Unifrac, Bray-Curtis, and Jaccard were 0.019, 0.018, and 0.016, respectively, indicating significant differences between the two groups. Next, we investigated changes in the abundance of microbial taxa at the genus level, as well as performed LefSe and random forest analyses. We analyzed the changes in the abundance of microbial taxa at the genus level between the two groups of mice. After KAR treatment, the relative abundances of *Lactobacullus*, *Bacteroides*, *Pelomonas*, and *Turicibacter* significantly increased, while the relative abundances of Ruminococcaceae UCG-014, Candid (**Figure 4B**). Moreover, we further explored the microbial species that exhibited significant differences after KAR treatment using LefSe and random forest analyses. After KAR treatment, the abundance of *Lactobacillus* genus, Ruminococcaceae_UCG_003 genus, Lactobacillales order, Lactobacillaceae family, and Bacilli class significantly increased, while the abundance of Mollicutes class, Mollicutes_RF39 order, and others significantly decreased (**Figure 4C**). KAR intervention significantly increased the relative abundances of beneficial microbial taxa such as *Lactobacullus* and *Ruminiclostridium*, while significantly decreasing the relative abundances of Ruminococcaceae UCG-014 and Candidatus Saccharimonas (**Figure 4D**), indicating that KAR significantly reduced the abundance of harmful microbial taxa such as Mollicutes and Mollicutes_RF39, while restoring the abundance of beneficial microbial taxa (Lactobacullus, Lactobacillales, and Lactobacillaceae).

### KAR ameliorates colitis via regulating T cell-mediated immunity

**Figure 5:**
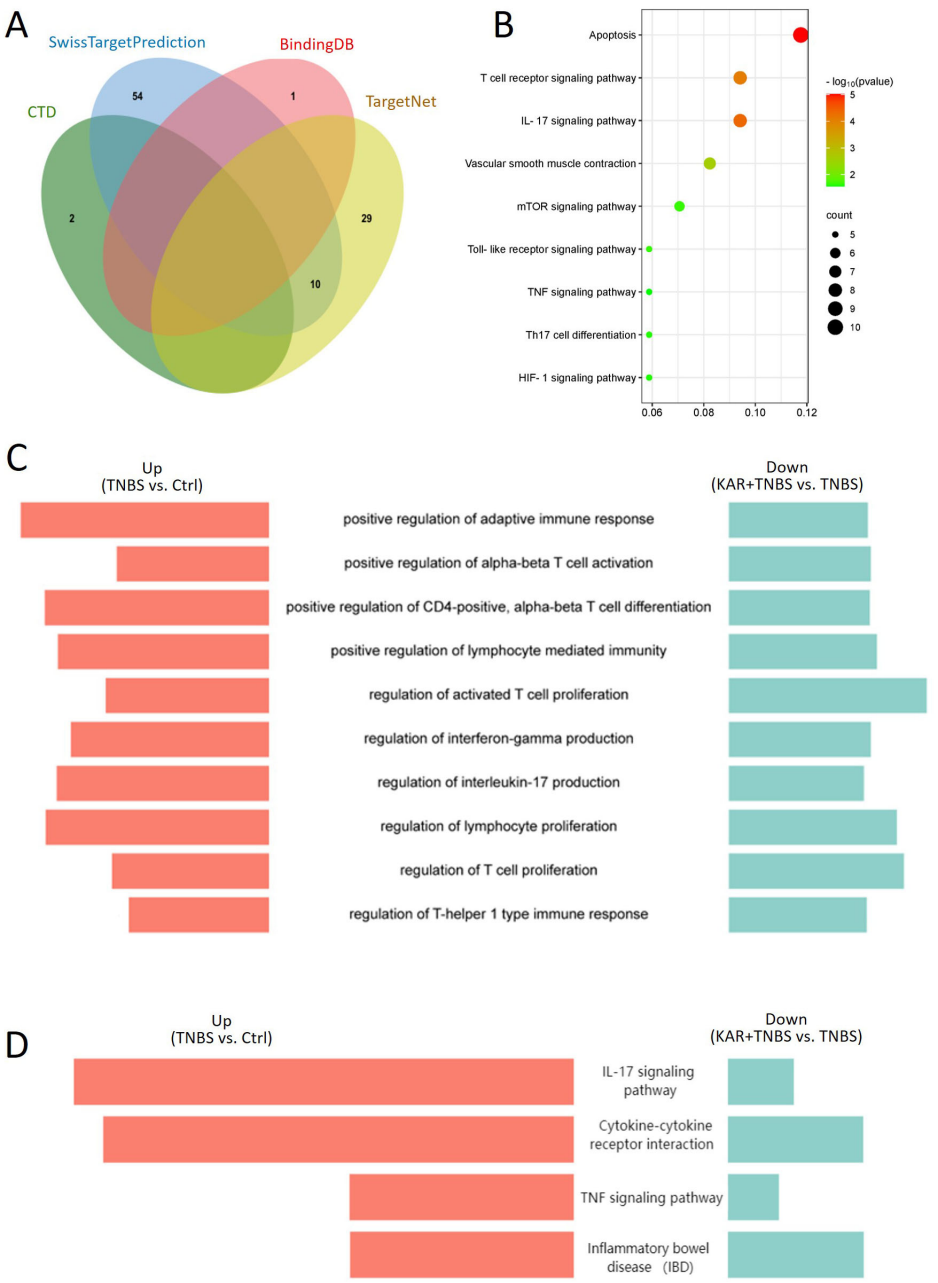
KAR mediates T cell immunity. **(A)** Venn diagram of predicted target genes for KAR obtained from the databases CTD, SwissTargetPrediction, BindingDB, and TargetNet. **(B)** KEGG pathway enrichment analysis of the predicted target genes. **(C, D)** Comparative analysis of upregulated genes in the TNBS group versus the control group, and downregulated genes in the KAR+TNBS group versus the TNBS group. The overlapped genes were subjected to **(C)** GO and **(D)** KEGG analysis. Enriched pathways are displayed as butterfly plots.

To further elucidate the underlying mechanisms by which KAR ameliorates colitis, we employed a combination of bioinformatics approaches and RNA sequencing. Initially, we identified proteins and pathways associated with KAR treatment through the integration of bioinformatics databases and experimental data. We queried the databases CTD, SwissTargetPrediction, BindingDB, and TargetNet to identify validated targets of KAR from previous studies and predicted potential signaling pathways based on the molecular structure of KAR (**Figure 5A**). Subsequent KEGG analysis of the predicted genes revealed significant enrichment in pathways related to T-cell immunity, including the IL-17 signaling pathway and Th17 cell differentiation, suggesting that T-cell-mediated inflammatory responses might be a potential target of KAR (**Figure 5B**). Next, we investigated the changes in gene expression within the colonic mucosa of colitis mice following KAR treatment using transcriptome sequencing. To identify genes associated with KAR treatment, we performed a comparative analysis of upregulated genes in the TNBS group versus the control group, and downregulated genes in the KAR+TNBS group versus the TNBS group. The overlapped genes were subjected to GO (**Figure 5C**) and KEGG (**Figure 5D**) analysis, which revealed significant enrichment in pathways related to T-cell responses, such as the regulation of IL-17 production, cytokine-cytokine receptor interaction, and IBD. Combined with the bioinformatics predictions, these findings indicated that Th17 cell responses could be a potential target of KAR in IBD.

**Figure 6:**
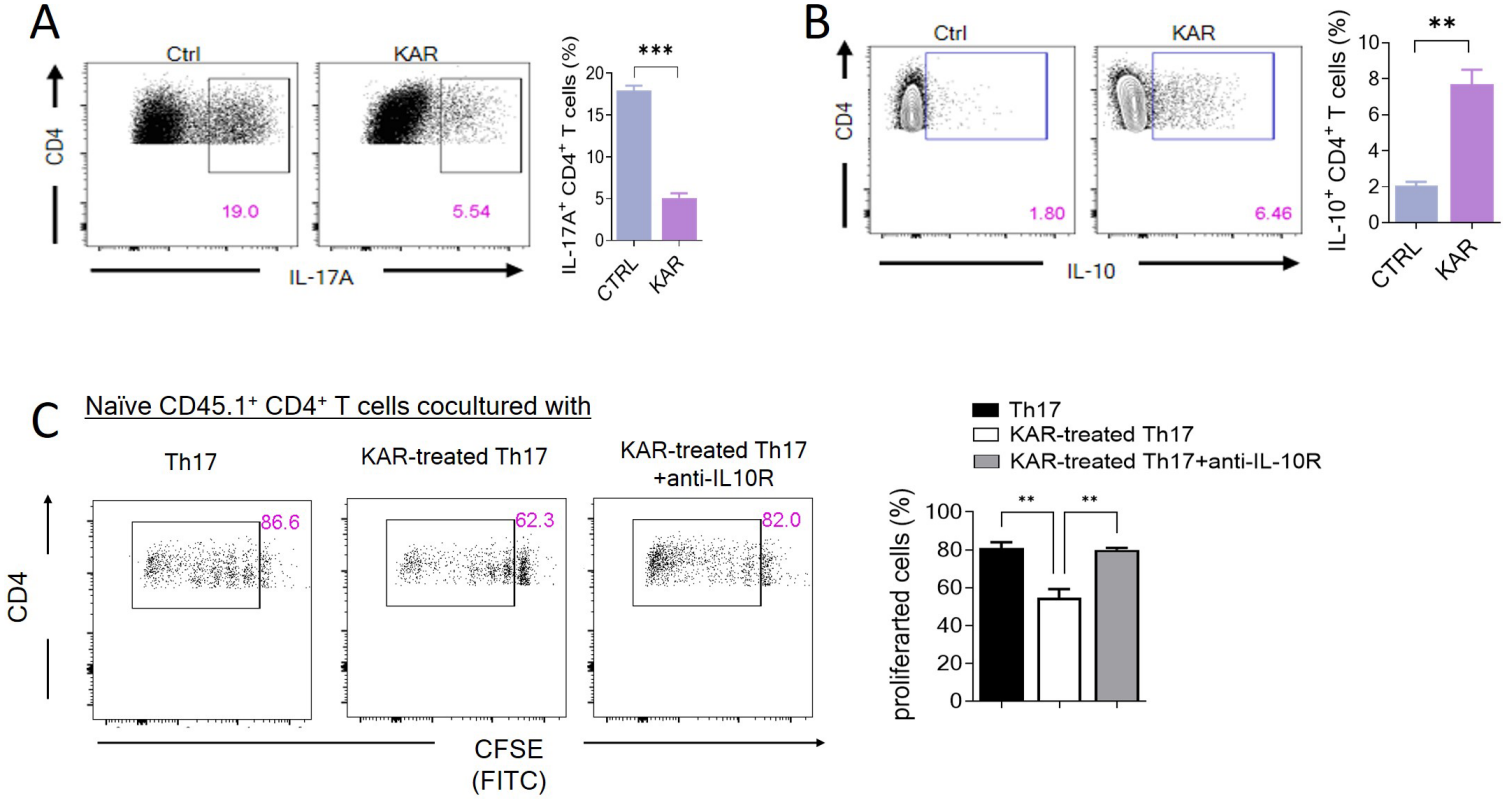
KAR facilitates T helper (Th)17 cell production of interleukin (IL)-10. Splenic CD4^+^ T cells were purified and differentiated under Th17-skewing conditions with or without KAR for 5 days. After harvesting, flow cytometry was performed to analyze CD4^+^ T cell expression of **(A)** IL-17A and **(B)** IL-10. The frequencies of IL-17A^+^ and IL-10^+^ CD4^+^ T cells are shown as a histogram. Unpaired Student’s t test, ** *p* < 0.01, *** *p* < 0.001, compared to cells without KAR treatment (ctrl). **(C)** Splenic CD4^+^ T cells from CD45.2 mice were purified and differentiated under Th17 conditions with or without KAR for 5 days. These cells were then cocultured with carboxyfluorescein succinimidyl ester (CFSE)-labeled splenic naïve CD4^+^ T cells from CD45.1 mice for 5 days. Anti-IL-10 receptor (IL-10R) antibody was added to block the IL-10 pathway. Flow cytometry was performed to analyze the CFSE intensity (FITC). The frequencies of proliferated cells are shown as a histogram. One way ANOVA, ** *p* < 0.01 compared to naïve CD45.1 cells cocultured with Th17 or KAR-treated Th17 cells plus anti-IL-10R.

To further confirm whether KAR modulated Th17 cell immunity, we performed *in vitro* cell culture experiments, demonstrating that KAR could significantly inhibit the differentiation of T cells into Th17 cells. Our previous studies have shown that when Th17 differentiation is inhibited (**Figure 6A**), the expression of the anti-inflammatory cytokine IL-10 is upregulated (**Figure 6B**), which is an essential regulatory mechanism for T-cell-mediated mucosal inflammation in IBD (21). Furthermore, *in vitro* inhibition assays confirmed that KAR-treated Th17 cells could exert immunosuppressive effects through IL-10 secretion (**Figure 6C**). Taken together, KAR ameliorates colitis by inhibiting the differentiation of Th17 cells and upregulating IL-10 secretion, thereby suppressing mucosal inflammation in IBD.

### KAR mediates IL-10 expression in Th17 cells via Blimp-1

**Figure 7:**
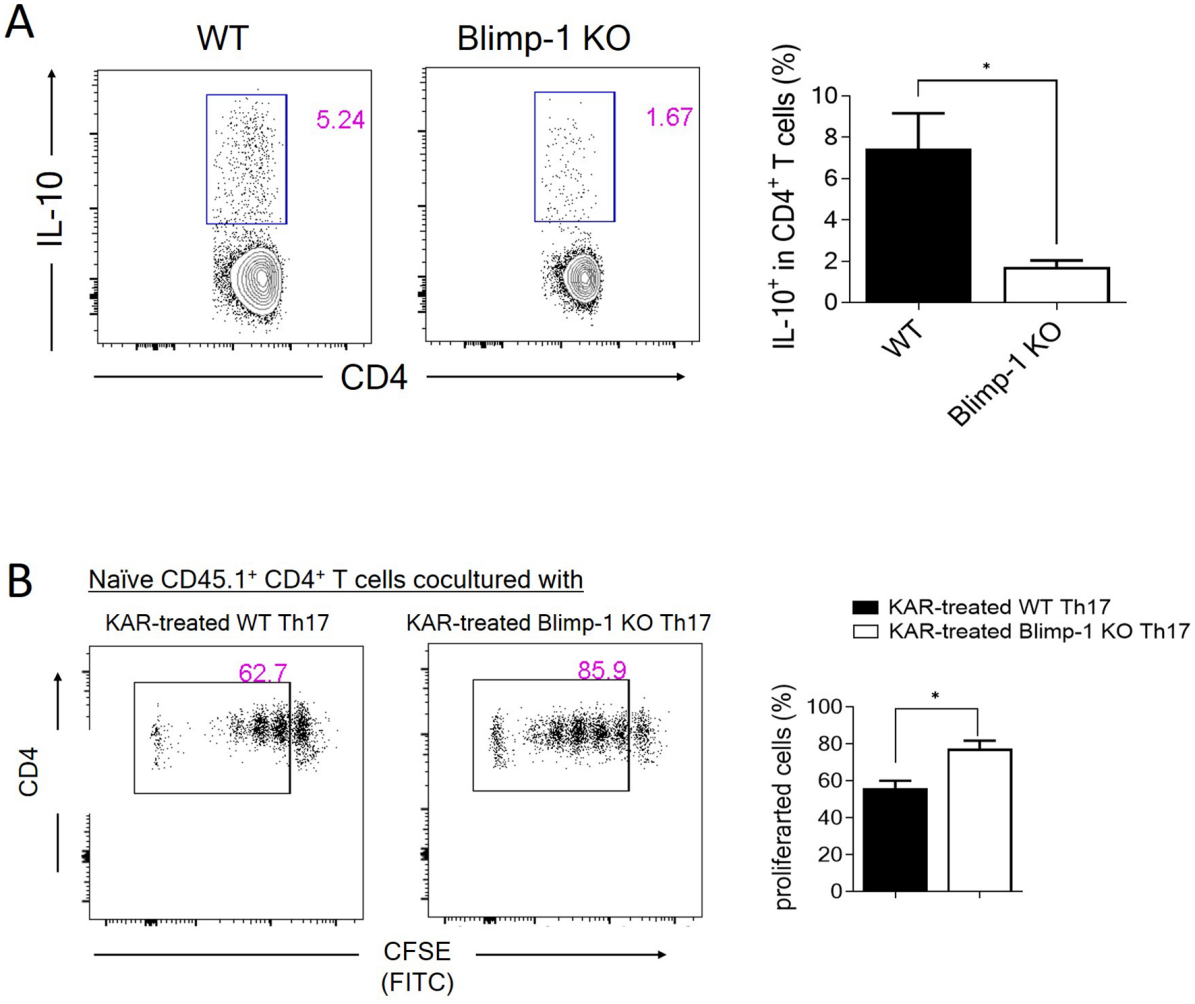
KAR mediates IL-10 expression in Th17 cells via Blimp-1. **(A)** CD4^+^ T cells were purified from wild type (WT) and Blimp-1 knockout (Blimp-1 KO) mice and differentiated under Th17 conditions with or without KAR. CD4^+^ T cell expression IL-10 was measured by flow cytometric intracellular staining. The frequencies of IL-10^+^ CD4^+^ T cells are shown as a histogram. **(B)** WT and Blimp-1 KO CD4^+^ T cells were purified and differentiated under Th17 conditions with or without KAR for 5 days. Two groups of cells were then cocultured with CFSE-labeled CD45.1^+^ naïve CD4^+^ T cells, respectively. Flow cytometry was performed to analyze the CFSE intensity (FITC). The frequencies of proliferated cells are shown as a histogram. Unpaired Student’s t test, * *p* < 0.05.

To further explore the molecular mechanisms underlying KAR-mediated IL-10 production in Th17 cells, we focused on Blimp-1, which has been shown to be crucial for IL-10 expression in Th17 cells in both previous studies and our own research (25–27). Therefore, we investigated whether KAR promotes IL-10 production in Th17 cells through Blimp-1. We used Blimp-1 KO mice and their wild type (WT) littermates to isolate CD4^+^ T cells from the spleen. These cells were then treated with KAR and induced to differentiate into Th17 cells. Flow cytometry analysis revealed that after KAR treatment, the proportion of IL-10-positive CD4^+^ T cells in Blimp-1 KO mice was significantly reduced compared to that in WT mice (**Figure 7A**). Additionally, the inhibitory effect of KAR-treated Th17 cells on T cell proliferation (**Figure 6C**) was impaired by Blimp-1 deficiency (**Figure 7B**). Taken together, these findings indicate that Blimp-1 is a key mediator of KAR-induced IL-10 expression in Th17 cells.

## Discussion

In this study, we observed that treatment with KAR significantly ameliorated colitis in mice, as evidenced by milder clinical symptoms and reduced colonic tissue damage. Specifically, KAR treatment led to a notable decrease in the infiltration of inflammatory cells, including monocytes/macrophages, neutrophils, and T lymphocytes, particularly reducing the pro-inflammatory subtypes of monocytes/macrophages and dendritic cells. Regarding the intestinal epithelial barrier, KAR treatment resulted in a mild reduction in goblet cells within the colonic epithelium but a significant upregulation of mucin expression compared to the TNBS group. Additionally, KAR treatment increased the expression of tight junction proteins such as Occludin, E-cadherin, and ZO-1, suggesting enhanced intestinal barrier integrity. Furthermore, KAR treatment restored the gut microbiota towards a normal composition. Mechanistically, KAR not only suppressed Th17 cell differentiation, but also upregulated their production of IL-10 via Blimp-1. These findings collectively suggest that KAR has significant potential as a therapeutic agent for IBD.

TCM, a treasure trove of medical wisdom, has garnered increasing attention in modern medical research. Modern pharmacological studies have demonstrated that TCM exerts therapeutic effects through the synergistic actions of multiple components, pathways, and targets. Herbal treatments often possess multiple active compounds with synergistic actions, targeting various pathological pathways involved in IBD (28). Additionally, herbal medicines are generally well-tolerated and may offer a lower risk of adverse effects compared to conventional pharmaceuticals. Despite the complex nature of herbal medicines, advancements in research methodologies have enabled the identification and characterization of their active ingredients, mechanisms of action, and potential therapeutic benefits. In this context, KAR has emerged as a promising compound with demonstrated efficacy in reducing inflammation and ameliorating colitis symptoms. Further research is warranted to fully elucidate the therapeutic potential of KAR in IBD.

The intestinal mucosal barrier is a complex and dynamic system composed of epithelial cells, tight junction complexes, mucus layers, secretory immunoglobulin A (sIgA), antimicrobial peptides (AMPs), and Paneth cells. Under physiological conditions, this barrier maintains a dynamic equilibrium, with stem cells continuously proliferating to replace shed epithelial cells, thereby preserving the integrity of the epithelial layer. Tight junction proteins such as ZO-1, Occludin, Claudin, and E-cadherin form a robust seal between intestinal epithelial cells. Additionally, goblet cells secrete mucins, particularly Mucin2, which provides an extra protective gel-like mucus layer for the epithelial cells. In IBD, however, many of these components fail, leading to disrupted tight junctions, reduced mucus production by goblet cells, and downregulated mucosal repair capacity. As a result, the intestinal epithelium becomes more permeable (29). Studies have shown that intestinal mucosal barrier dysfunction may occur in the early stages of IBD. For instance, specific defects in intestinal epithelial cells can induce colitis even in the presence of normal immune function and gut microbiota (30). Conversely, pharmacological enhancement of the intestinal mucosal barrier function can alleviate intestinal inflammation in mice (31). These findings highlight that a complete, microbe-impermeable mucosal barrier is essential for maintaining intestinal homeostasis, and improving intestinal mucosal barrier function is an important therapeutic target for UC. Li et al. reported that KAR promotes sIgA secretion, thereby enhancing the regulation of the intestinal mucosal barrier and improving resistance to pathogens (32). In an irritable bowel syndrome (IBS) mouse model, KAR effectively alleviated visceral hypersensitivity and maintained intestinal barrier function (12). In our study, we observed severe disruption of colonic epithelium in TNBS-treated mice, with significant reductions in goblet cells and mucin, as well as decreased expression of tight junction proteins Occludin, ZO-1, and E-cadherin. Following KAR treatment, TNBS-induced mice exhibited better-preserved epithelial integrity, improved goblet cell preservation, enhanced mucus secretion, and increased expression of Occludin, ZO-1, and E-cadherin in colonic tissues. These results suggest that KAR has the potential to repair intestinal mucosal barrier function and offers protective effects on multiple aspects of the intestinal barrier.

The abnormal response of CD4^+^ T cells to the gut microbiota is a central mechanism in the pathogenesis of IBD. In this context, Th17 cells, a subset of CD4^+^ T cells producing IL-17, have been closely associated with the progression of IBD. Recent studies on the IL-23/Th17 axis have shown that Th17 cells dominate in the gut of chronic experimental colitis mice (33). Compared to healthy controls, patients with active IBD exhibit significant infiltration of Th17 cells in the intestinal mucosa, with elevated expression of Th17-related cytokines (including IL-17, IL-21, and IL-22) at inflammatory sites and in peripheral blood. These cytokines are positively correlated with disease severity in IBD (34). Furthermore, transferring Th1 and Th17 cells into severe combined immune deficiency (SCID) mice revealed that while Th1 cells induced mild chronic colitis, Th17 cells caused severe intestinal mucosal damage (33). These findings highlight the critical role of Th17 cells in the immunopathology of IBD. In our study, we observed that KAR significantly downregulated Th17 cells in the colonic mucosa of TNBS-induced colitis mice. Consistent with our findings, previous studies have shown that KAR inhibits Th17 cell differentiation and proliferation in the experimental autoimmune encephalomyelitis (EAE) mouse model, reducing the expression of multiple pro-inflammatory cytokines and preventing the infiltration of inflammatory cells into the central nervous system (13). Similarly, Tang et al. reported that KAR treatment significantly reduced the levels of IL-17A and other pro-inflammatory cytokines in the serum and paw tissues of collagen-induced arthritis (CIA) mice (35). These results collectively support the notion that KAR modulates CD4^+^ T cell responses, thereby attenuating inflammation.

Interestingly, KAR treatment not only downregulated the secretion of IL-17A by Th17 cells but also increased the expression of IL-10. The inhibitory effect of KAR on Th17 cell proliferation was abolished upon IL-10 blockade, suggesting that IL-10 is crucial for KAR-mediated regulation of Th17 cells. IL-10, a key anti-inflammatory cytokine, is essential for the immunosuppressive function of regulatory T cells (Tregs) in IBD and for maintaining mucosal homeostasis in both effector CD4^+^ T cells and innate immune cells. Li et al. demonstrated that KAR downregulated IL-17A while upregulating TGF-β1 and IL-10, restoring the balance between Th17 and Treg cells and promoting intestinal immune homeostasis in UC (36). These findings align with our results and further highlight the importance of IL-10 in KAR-mediated immunomodulation. Notably, while CD4^+^ Tregs are the primary source of IL-10, effector CD4^+^ T cells can also transiently co-express IL-10 as part of an intrinsic negative feedback mechanism to limit inflammatory immune responses (37). This suggests that KAR may enhance the anti-inflammatory properties of Th17 cells by promoting IL-10 expression, thereby contributing to the resolution of intestinal inflammation.

Blimp-1, encoded by the *Prdm1* gene, is a multifunctional transcriptional regulator involved in the modulation of a wide range of genes associated with cell signaling, communication, and survival (38, 39). Previous studies have highlighted the critical role of Blimp-1 in the expression of the immunoregulatory cytokine IL-10 (40). For instance, in the context of IBD, Blimp-1-mediated IL-10 production by CD4^+^ T cells has been demonstrated to suppress colitis (25, 27). Moreover, genetic variations in *Prdm1* gene have been linked to the susceptibility of IBD (41), further emphasizing the importance of Blimp-1 in immune regulation. In our study, we observed a significant reduction in IL-10-positive CD4^+^ T cells in Blimp-1 KO mice compared to WT mice following KAR treatment. This finding supports that KAR promotes IL-10 production in Th17 cells at least partly through Blimp-1. This mechanism appears to be crucial for the immunoregulatory effects of KAR in intestinal mucosal inflammation. Given the established role of Blimp-1 in driving the differentiation of immunosuppressive cells and modulating cytokine production, our results suggest that KAR may enhance the regulatory functions of Th17 cells by upregulating Blimp-1, thereby contributing to the resolution of inflammation and maintenance of mucosal homeostasis. Future research should focus on elucidating the specific signaling pathways through which KAR activates Blimp-1 in Th17 cells and exploring the therapeutic potential of targeting this pathway in inflammatory diseases.

Despite the promising findings, this study has several limitations. Firstly, the research was conducted in a mouse model of colitis, which may not fully represent the complexity of human IBD. Therefore, caution should be exercised when extrapolating the results to human patients. Secondly, the mechanisms underlying the effects of KAR on various aspects of IBD pathogenesis require further elucidation. Future studies should aim to explore these mechanisms in more detail. Additionally, long-term effects and potential side effects of KAR treatment need to be investigated to ensure its safety and efficacy as a therapeutic option for IBD.

In conclusion, this study highlights the potential of KAR as a therapeutic agent for IBD. Although additional studies are needed to address the limitations of this research and fully elucidate the mechanisms underlying the therapeutic effects of KAR, our findings contribute to the growing body of knowledge on IBD pathogenesis and offer promising perspectives for the development of novel therapeutic approaches.

## Data Availability

The raw data supporting the conclusions of this article will be made available by the authors, without undue reservation.
